# Urbanization alters interactions between Darwin's finches and *Tribulus cistoides* on the Galápagos Islands

**DOI:** 10.1002/ece3.8236

**Published:** 2021-10-26

**Authors:** L. Ruth Rivkin, Reagan A. Johnson, Jaime A. Chaves, Marc T. J. Johnson

**Affiliations:** ^1^ Department of Ecology and Evolutionary Biology University of Toronto Toronto Ontario Canada; ^2^ Department of Biology University of Toronto Mississauga Ontario Canada; ^3^ Centre for Urban Environments University of Toronto Mississauga Ontario Canada; ^4^ St. James Catholic Global Learning Centre Mississauga Ontario Canada; ^5^ Colegio de Ciencias Biológicas y Ambientales Universidad San Francisco de Quito Quito Ecuador; ^6^ Department of Biology San Francisco State University San Francisco California USA

**Keywords:** anthropocene, herbivory, Jamaican feverplant, pinzon, plant defense, plant‐herbivore, puncture vine, seed predation, urban ecology, urban evolution

## Abstract

Emerging evidence suggests that humans shape the ecology and evolution of species interactions. Islands are particularly susceptible to anthropogenic disturbance due to the fragility of their ecosystems; however, we know little about the susceptibility of species interactions to urbanization on islands. To address this gap, we studied how the earliest stages of urban development affect interactions between Darwin's finches and its key food resource, *Tribulus cistoides*, in three towns on the Galápagos Islands. We measured variation in mericarp predation rates, mericarp morphology, and finch community composition using population surveys, experimental manipulations, and finch observations conducted in habitats within and outside of each town. We found that both seed and mericarp removal rates were higher in towns than natural habitats. We also found that selection on mericarp size and defense differed between habitats in the survey and experimental populations and that towns supported smaller and less diverse finch communities than natural habitats. Together, our results suggest that even moderate levels of urbanization can alter ecological interactions between Darwin's finches and *T*. *cistoides*, leading to modified natural selection on *T*. *cistoides* populations. Our study demonstrates that trophic interactions on islands may be susceptible to the anthropogenic disturbance associated with urbanization. Despite containing the highest diversity in the world, studies of urbanization are lacking from the tropics. Our study identified signatures of urbanization on species interactions in a tropical island ecosystem and suggests that changes to the ecology of species interactions has the potential to alter evolution in urban environments.

## Graphical abstract

Urban evolutionary ecology is a rapidly expanding field and yet is understudied in the tropics and on islands. We found strong evidence that, even at its earliest stages, urbanization can alter ecological interactions between Darwin's finches and *Tribulus cistoides*, leading to modified phenotypic selection on *T. cistoides* populations. Our study is one of the first to identify urbanization as a key mediator of species interactions on a tropical island archipelago.

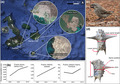

## 1 INTRODUCTION

Urbanization results in substantial changes to the environment. Urban habitats are typically warmer, more polluted, and more fragmented than nearby non‐urban habitats, which can lead to changes in the abundance and persistence of populations, as well as altered diversity and community composition (Grimm et al., 2008; Niemelä, 2011; Seto et al., 2012). Emerging evidence suggests that ecological changes associated with urbanization may lead to selection for novel adaptations (Johnson & Munshi‐South, 2017; Szulkin et al., 2020). Urbanization also affects interactions among species; however, it is difficult to predict how species interactions will respond to urbanization (Miles et al., 2019; Vincze et al., 2017). Trophic interactions are inherently interconnected and may be susceptible to urbanization through effects on one or both trophic levels. Urbanization may decouple predator–prey interactions through the addition of food subsidies from anthropogenic sources (Rodewald et al., 2015) or may intensify interactions when urban habitat fragmentation reduces available niche space, forcing species to interact more frequently (Magle et al., 2014; Miller et al., 2017). Additionally, urbanization may act to filter urban communities, preventing some species from occupying the habitat while promoting the abundance of more resilient or tolerant species (Aronson et al., 2016). Together, these changes have the potential to lead to novel selection pressures on one or both sets of interacting species (Miles et al., 2019), although it is unclear how frequently urbanization results in altered natural selection on interacting species.

Most examples of contemporary urban ecology and evolution occur in well‐established cities, especially in Europe and North America (Gorton et al., 2020; Johnson & Munshi‐South, 2017; Rivkin et al., 2019). We currently have limited knowledge of how urbanization in tropical regions, and particularly on tropical islands, can influence the ecology and evolution of species. Islands may be particularly sensitive to urbanization because of the unique and fragile ecosystems they support. Island ecosystems often exist within a narrow range of environmental conditions, have simplified community structure, and are known to be sensitive to disturbance to the environment (Hadfield et al., 1993; Whittaker & Fernández‐Palacios, 2007). Due to this sensitivity, even small human settlements may have large effects on communities (Graham et al., 2017; Helmus et al., 2014). Urban development is increasing on many islands (Brunn et al., 2011), and human actions and decision‐making that are both deliberate (e.g., urban planning and zoning regulation) and unintended (e.g., introduction of human pets as predators and increased food subsidies due to waste management) can influence ecological and evolutionary processes on islands by altering the structure and functioning of islands communities. Our study tests questions related to urbanization on island evolutionary ecology using the Darwin's finch–*Tribulus* interaction of the Galápagos archipelago.

The Galápagos Islands of Ecuador provide an ideal system to test questions about how urban development affects species interactions on islands. Ground finches (*Geospiza* spp.) and *Tribulus cistoides* L. (Zygophyllaceae, common names puncture vine or Jamaican feverplant) on the Galápagos have a long history of study (Grant, 1999; Grant & Grant, 2014; Lack, 1947), and recent evidence suggests that these species are experiencing an ongoing co‐evolutionary arms race (Carvajal‐Endara et al., 2020). *Tribulus cistoides* is an important food resource for three medium‐ and large‐beaked ground finch species: *Geospiza fortis*, *G*. *magnirostris*, and *G*. *conirostris*, although not all species are present on every island of the Galápagos archipelago (Boag & Grant, 1984a; Grant & Grant, 1982; Grant, 1981). Predation on *T*. *cistoides* has led to niche segregation and adaptation in beak morphology in these finch species (Boag & Grant, 1981, 1984b; Grant & Grant, 2006). In turn, finches influence mericarp survival and select for smaller, harder, and more defended mericarps (Carvajal‐Endara et al., 2020). Anthropogenic effects on the Galápagos Islands influence both finch and *T*. *cistoides* populations (De León et al., 2019; Gotanda, 2020; Harvey et al., 2021; McNew et al., 2017). Humans are a key disperser of *T*. *cistoides* on the Galápagos (Johnson et al., 2020), and resource partitioning in *G*. *fortis* declines in towns (De León et al., 2019; Hendry et al., 2006), likely due to the increased availability of human food (De León et al., 2019) and human‐induced behaviour modifications (Gotanda, 2020).

**FIGURE 1:**
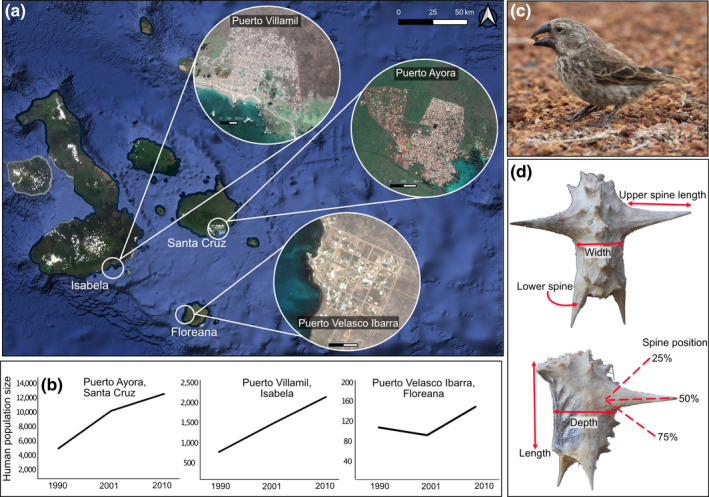
(a) Map of the Galápagos Islands, with the three islands sampled and their principal towns. Maps were taken from Google Satellite dating from 2018. (b) Change in population size in each town from 1990–2010 (INEC 2010), ordered from largest (Santa Cruz) to smallest (Floreana); note, the human population has continued to grow rapidly but censuses on all three islands are carried out only every 10 years. (c) A female medium ground finch (*Geospiza fortis*) holding a *Tribulus cistoides* mericarp in its beak. (d) Dorsal and lateral images of a *T*. *cistoides* mericarp, with each of the six morphological traits measured. Images in C and D taken by M.T.J.J

Despite clear evidence of anthropogenic effects on finches and *T*. *cistoides* individually, no work has examined how anthropogenic activity affects interactions between the two species. Our objective was to identify how towns at an early stage of urbanization affect interactions between ground finches and *T*. *cistoides*. We studied seed and mericarp predation, phenotypic selection on mericarp morphology, and finch community composition in towns and natural habitats on three islands on the Galápagos archipelago (Figure 1). We chose this multifaceted design to investigate how predation and phenotypic selection vary in different habitats under natural conditions (i.e., natural populations of *T*. *cistoides*) and in artificially simplified environments (i.e., experimental populations). We also aimed to investigate if potential changes in predation and phenotypic selection were associated with differences in the finch communities between habitats. Thus, our design allows us to rigorously test the effects of urbanization on both sides of the finch‐*Tribulus* interaction. We used this system to ask three specific questions: (1) Does mericarp predation by finches differ between towns and natural habitats of *T*. *cistoides*? (2) Does phenotypic selection imposed by seed predation on mericarp size and defense differ between habitats, as measured as the covariance between the rate of seed or fruit predation and mericarp morphology? (3) Does the Galápagos finch community differ between habitats? Here, we aim to identify the effects of the moderate urbanization on the evolutionary ecology of species interactions in an iconic island ecosystem.

## 2 MATERIALS AND METHODS

### 2.1 Study site and system

The Galápagos Islands are an archipelago 1,000 km off the coast of Ecuador (Geist, 1996). We studied the effects of urban development on three of the five inhabited islands in the Galápagos: Floreana, Isabela, and Santa Cruz (Figure 1a). These islands contain towns that differ in area and human population sizes, from 145 people inhabiting 3 km^2^ in Puerto Velasco Ibarra on Floreana to 2,300 people inhabiting 6 km^2^ in Puerto Villamil in Isabela, and 15,700 people inhabiting 10 km^2^ in Puerto Ayora on Santa Cruz (INEC, 2010, 2015). The human population has experienced rapid growth in the last 30 years (Figure 1b), resulting in substantial urban development (Benítez et al., 2018). Although Ecuador places strong environmental regulations on the Galápagos Islands, socio‐economic strategies, infrastructure growth, and waste management plans have been put in place to cope with the influx of tourists to the islands (Pizzitutti et al., 2017). The growth of urban development has impacted the availability of habitat for native flora and fauna, such as *T*. *cistoides* and Darwin's finches. In particular, *T*. *cistoides* is most common along roadsides, paths, the margins of parks, and beaches, all areas where people frequent.

The Galápagos Islands are home to a variety of ground finches (*Geospiza* spp. Gould) that have undergone an adaptive radiation (Grant & Grant, 1982, 2006; Lamichhaney et al., 2015). There are 11 species of ground finches present on the islands (Lamichhaney et al., 2015), including two of interest to our study: the medium ground finch (*G*. *fortis*) and the large ground finch (*G*. *magnirostris*). Both species are known to feed on *T*. *cistoides*, although *G*. *magnirostris* are more adept at extracting seeds (Grant, 1981). Both species are found on Isabela and Santa Cruz, but only *G*. *fortis* is present on Floreana. *Geospiza fortis* and *G*. *magnirostris* are relatively large finches, with large, strong beaks (Figure 1c). Beak morphology is highly correlated with diet; large‐beaked finches are capable of consuming larger and harder seeds than small‐beaked finches (Abbott et al., 1977). Diet is also associated with temporal variation in seed availability. During wet years, finches feed on seeds from a variety of species; however in dry years, food becomes scarce and large‐beaked finches feed mostly on hard seeds, such as those produced by *T*. *cistoides* (Grant & Grant, 1980).

*Tribulus cistoides* is widely distributed across tropical continents and islands, including on the Galápagos Islands (Porter, 1971). *Tribulus cistoides* is an herbaceous perennial that grows in arid lowland and coastal habitats (Schweickerdt, 1868) and is often found in sand or volcanic soil alongside roads, trails, and beaches in both town and natural habitats on the Galápagos Islands (Johnson et al., 2020). Most vegetative growth of *T*. *cistoides* occurs during the rainy season, followed by a prolonged flowering period (Porter, 1971). *Tribulus cistoides* produces hard fruits, which separate into five segments called mericarps. Mericarps are produced in the wet season and typically persist for many months until germinating or decaying the following wet season (Porter, 1971). Each mericarp is defended by 0–4 sharp spines that serve as a defense against predation (Carvajal‐Endara et al., 2020; Grant, 1981) and as a mode of seed dispersal (Johnson et al., 2020). Each mericarp contains 1–7 seeds, which can be accessed by cracking open the mericarp, a feat that is difficult for all but the largest‐beaked birds (Grant, 1981). To crack open a mericarp, a bird picks it up and applies pressure with its beak, shearing off the ventral side of the mericarp wall and exposing the seeds (Carvajal‐Endara et al., 2020). This process leaves characteristic damage to mericarps that can be reliably identified months later. In addition to dispersal from finch predation, human‐assisted dispersal of *T*. *cistoides* fruits is common on the Galápagos (Johnson et al., 2020). Dispersal occurs at a higher rate in towns than natural habitats and occurs when the fruits become stuck to the bottom of shoes, car tires, and on various fabrics (Johnson et al., 2020).

### 2.2 Study design

This study was comprised of three parts: a survey of populations of *T*. *cistoides* to estimate seed removal; an experiment with mericarp defense traits artificially manipulated to measure mericarp removal; and finch community observations. We studied each of these components at the beginning of the wet season, from January–March 2018 in towns and natural habitats on each of the three islands. For each component described below, we considered a habitat to be in a town if it occurred within a town's borders and natural if it grew outside a town's borders, which are easily determined by the lack of densely spaced housing.

#### 2.2.1 Population survey

We conducted a survey of seed removal from *T*. *cistoides* populations in February 2018 to test for differences in seed removal rates and phenotypic selection among towns and natural habitats. These populations provided us with a picture of variation in seed removal between towns and natural habitats across islands over an 8‐ to 12‐month period, which is how long mericarps persist once dehiscing (Porter, 1971). We defined each population as a group of *T*. *cistoides* plants growing together with at least 100 m separation from the next nearest grouping. We sampled 16 populations on Floreana (*N* = 9 town and 7 natural), 28 populations on Isabela (*N* = 15 town and 13 natural), and 41 populations on Santa Cruz (*N* = 22 town and 19 natural). We collected 20 mericarps that were lying on the ground from each population (except for one population where we found only 19 mericarps), for a total of 1,699 mericarps. We counted the number of seeds missing from each mericarp to estimate seed removal by finches. It is possible to count missing seeds because each seed is housed in a locule in the mericarp, making it clear how many seeds were originally contained in the mericarp even after they have been removed. Following the protocol outlined in Carvajal‐Endara et al. (2020), we measured six morphological traits on each mericarp: mericarp length, width, depth, the length of the longest spine, presence or absence of lower spines, and spine position (Figure 1d). We used these trait measurements to generate composite variables for mericarp size and defense (described in the *Statistical Analyses* section).

#### 2.2.2 Fruit removal experiment

At the same time as the population surveys, we conducted a 6‐week‐long experiment to test for variation in fruit removal and phenotypic selection in *T*. *cistoides*. This experiment complemented our population surveys by allowing us to causally determine how morphology affects removal and phenotypic selection on mericarps by finches. On each island, we collected 800–900 intact mericarps (i.e., not attacked by finches) from natural habitats. We weighed the mericarps and measured the same six morphological traits measured in the population survey (Figure 1d), from which we calculated new composite variables for size and defense (*Statistical Analyses*). We selected 20 town and 20 natural sites per island (*N* = 40 sites per island) and placed a petri dish (100 mm diameter) in each habitat in January 2018. Each dish contained 20 mericarps placed on top of locally collected substrate (i.e., volcanic sand and gravel) for a total of 2,120 mericarps. We randomly selected half of the mericarps and used wire cutters to clip off all their spines to create an “undefended” mericarp, while the other half were left with their spines intact as “defended” mericarps. We selected mericarps that had four spines so that our manipulation simulated fully defended (four spines) versus undefended (zero spines) mericarps. We marked each mericarp with a unique identifier on its dorsal surface using a black permanent marker so that we could identify each individual mericarp at the end of the experiment.

We left the mericarps in the field for 6 weeks, and then collected them to score removal. Using the identifying marks placed on the mericarps, we determined which mericarps had been removed and which remained in the tray. If a mericarp was removed, we counted it as “eaten” because ground finches often carry mericarps away from the location where they collect them to crack them on a hard surface. In this way, mericarp removal served as our proxy for fitness effects via seed consumption. We placed petri dishes in locations where humans would not walk, thus we are confident that mericarp removal was due to finch consumption and not human dispersal (Johnson et al., 2020). Several petri dishes were disturbed during the experiment (three on Floreana, six on Isabela, eight on Santa Cruz), so we excluded those dishes from the analyses.

#### 2.2.3 Finch community observations

To determine how ground finch community composition varies with urban development, we conducted surveys at town and natural *T*. *cistoides* populations on each island. We selected six sites per island (*N* = 3 town and 3 natural) ensuring that each habitat had clear lines of sight within 50 m of the center of the population. We conducted point counts at each location for 5 min, where we recorded finch sightings within 50 m during that time (Bibby et al., 1998). Although *G*. *fortis* and *G*. *magnirostris* are the only vertebrate seed predators of *T*. *cistoides* on the islands (Carvajal‐Endara et al., 2020), we recorded all finch species that frequently interact with *G*. *fortis* and *G*. *magnirostris* and thus could influence their distribution, abundance, or behavior. We repeated the surveys four times on Santa Cruz and three times on Floreana and Isabela.

### 2.3 Statistical analyses

We used R v. 3.6.2 (R Development Core Team, 2008) for all analyses.

#### 2.3.1 Population surveys

We generated composite variables for mericarp size and defense by running a principal component analysis (PCA) on the six morphological variables we measured (Table S1, Figure S1a). We first standardized and centered each variable to a mean of 0 and standard deviation of 1. The first principal component (PC) axis was associated with mericarp size and explained 45% of the variation in the data, and all variables loaded in the same direction on this axis (Figure S1a). The second PC axis was associated with defense and explained 20% of the variation in the data. Spine length, position, and presence of lower spine loaded in the same direction on this axis (Figure S1a). Based on these loadings, we used PC1 as a composite representative of size (hereafter: size) and PC2 as a composite representative of defense (hereafter: defense).

We tested for the differences in the number of seeds eaten per mericarp across populations using a Poisson‐distributed linear mixed‐effects model using the *glmmTMB* v. 1.0 package (Brooks et al., 2017). We set the model to account for zero inflation because more than half the mericarps sampled were completely intact. The model was constructed as follows:

$$\text{Number of seeds eaten per mericarp}∼Habitat\left[H\right]\;+Island\left[I\right]\;+Size\left[S\right]+Defense\left[D\right]\;+H:I+H:S+H:D+I:S+I:D+\left(1|I:Population\right)$$

The number of seeds eaten per mericarp was an integer that ranged from 0 to 5 and is a proxy for fitness, where more seeds eaten per mericarp results in lower fitness of the plant. Although this measure is not an estimate of relative fitness, we are able to infer the phenotypic selection pressure imposed by finch predation by the reduction in seeds that were eaten per fruit. *Habitat* and *Island* were categorical fixed effect variables with two levels (town and natural) and three levels (Floreana, Isabela, Santa Cruz), respectively. *Size* and *defense* were continuous fixed effect variables that corresponded to the first and second PC axes of mericarp morphology, respectively. Mericarps differ in the number of seeds they contain, with bigger mericarps containing more seeds (Carvajal‐Endara et al., 2020). By including mericarp size (PC1) in the model, we were able to account for differences in seed number due to size, allowing us to accurately interpret the effects of the other variables in the model. We incorporated population as a random effect in the model and allowed it to vary among islands. This allowed us to account for variation among populations due to differences between islands.

We tested two other models of seed predation: (1) a zero‐inflated hurdle model that uses a binomial component for the probability that a given observation is zero or not and a Poisson component modeling the number of observations for non‐zero values, and (2) a binomial model that includes seed removal as a binary (eaten or not) term. We compared the fit of each model using a likelihood ratio test and AIC values. The original, zero‐inflated Poisson model was a significantly better fit than either the hurdle or binomial models (*p* < .001); thus we proceeded to use this model in our analysis. Lastly, we used the *DHARMa* v. 1.4.1 package (Hartig, 2021) to confirm that there were no issues with the distribution of residuals.

We assessed the significance of fixed effects from both models using analysis of variance (ANOVA) implemented using the *Anova* function in the *car* v. 3.0‐6 package (Fox & Weisberg, 2019) to calculate Wald *χ* ^2^ test statistics with Type III sums‐of‐squares to test for significant interaction terms. Although Type II sums‐of‐squares are typically used in cases with incomplete or unbalanced datasets (Langsrud, 2003), we opted to assess the significance of our results with Type III sums‐of‐squares because we observed significant interaction terms that were related to our *a priori* hypotheses (Hector et al., 2010; Shaw & Mitchell‐olds, 1993). A significant main effect of habitat or island would indicate that these factors affect the number of seeds eaten per mericarp. A significant main effect of size and/or defense would indicate that these traits influence the number of seeds eaten per mericarp, suggesting that seed removal imposes phenotypic selection on these traits. A significant interaction between island and habitat would indicate that the effect of habitat on seed removal differs between islands. A significant interaction between habitat and size/defense would indicate that phenotypic selection on these traits differ between town and natural habitats. Lastly, a significant interaction between the island and size/defense would indicate that phenotypic selection on these traits by finches differs between islands.

Following this analysis, we tested for differences in fruit morphology between habitats and among islands. We ran six separate mixed‐effect generalized linear models with each morphological measurement (Figure 1d) as the response variable and habitat and islands as predictors. As before, we included population as a random effect to account for variation among populations on each island. We conducted an ANOVA on each of the models with adjusted *p*‐values to account for running multiple tests. Differences in trait values between habitats would suggest that the habitat alters trait values but does not allow us to determine if such differences are due to evolution or phenotypic plasticity.

#### 2.3.2 Fruit removal experiment

As with the population survey dataset, prior to analysis we used a PCA to generate composite variables for size and defense (Table S1, Figure S1). We included the same six morphological measurements measured on the mericarps collected for the experiment, along with mericarp mass. We extracted PC1 (size: 46% of the variation among variables) and PC2 (defense: 17% of the variation among variables) to use in our model; each additional PC axis explained <15% of the variation in morphology and was not used in subsequent analyses. These values differ slightly from the PC axes from the population survey analysis because they are extracted from a different set of mericarps. However, the loadings of the variables on PC1 and PC2 occur in the same direction and are of similar magnitude as those from the survey analysis, thus we are confident that they are capturing similar variation in size and defense.

We tested for differences in mericarp removal from the population using a binomial distributed logistic linear mixed‐effects model with a logit link function, implemented using the *glmer* function in *lme4*. The model was constructed as follows:

$$\text{Mericarp removal}∼Habitat\left[H\right]+Island\left[I\right]+Size\left[S\right]+Defense\left[D\right]+Clipped\left[C\right]+H:I+H:S+H:D+H:C+I:S+I:D+\left(1|I:Population\right)$$

Mericarp removal was categorized as a binary variable, where mericarps were recorded either being present in the dish (0, no predation) or having been removed from the dish (1, predation). *Habitat*, *island*, *size*, *defense*, and their interactions were treated and interpreted the same as in the survey analysis. *Clipped* was a categorical fixed effect factor with two levels (spines removed or intact). We assessed the assumptions of the model and tested for significance in the same manner as the survey analysis. A significant main effect term for the clipped treatment would indicate that artificially removing mericarp spines affects mericarp removal. A significant interaction term between *habitat* and *clipped* would indicate that the effect of spines on susceptibility to removal differs between town and natural habitats. Although the above model is complex, it is an extension of multivariate measures of phenotypic selection on survival data (Janzen & Stern, 1998).

#### 2.3.3 Finch community observations

We assessed variation in the ground finch community using multiple regressions and a constrained ordination analysis. We tested for differences in finch abundance using multiple generalized linear regression run on a Poisson distribution, and we tested for differences in diversity using a multiple linear regression model. We constructed the following model:

$$\text{Counts}/\text{diversity}∼Habitat\left[H\right]+Island\left[I\right]+Population+H:I$$

Counts was measured as the total number of birds observed at a population. Diversity was calculated as the Shannon Diversity Index, which measures species diversity by combining information on species richness and the evenness in relative abundance of species within a community. *Habitat*, *island*, and their interaction were the same as in the previous two analyses. Due to the small number of populations sampled, we were unable to run a mixed model with population as a random factor. Instead, *population* was included as a categorical fixed‐effect variable used to account for repeated sampling of populations. We determined that the distribution of residuals was normal and that there was no evidence of multicollinearity among main effects. We assessed the significance of the fixed effects using a Wald *χ* ^2^ test statistics with Type III sums‐of‐squares to test for significant interaction terms.

We estimated differences in community composition between town and natural habitats using redundancy analysis (RDA). An RDA combines multivariate ordination of a species composition matrix as the response variable with a multiple linear regression of predictor variables, which are fit using a PCA (Legendre & Legendre, 2012). The result is a series of canonical axes that can be tested for significance using a constrained canonical ANOVA (Legendre & Legendre, 2012). We conducted the RDA using the *rda* function in the *Vegan* v. 2.5‐6 package (Oksanen et al., 2020). The response matrix included species abundance observations with each population listed on a separate line. We transformed the species abundance matrix using a chord transformation to center and standardize the observations (Oksanen et al., 2020). The explanatory matrix included the habitat (town and natural), island, and population. Each line in the explanatory matrix contained data for the population that corresponded to the same line in the response matrix. We tested the significance of the effects using the *anova*.*cca* function in *Vegan*, with 1000 permutation cycles.

## 3 RESULTS

### 3.1 Population survey

**FIGURE 2:**
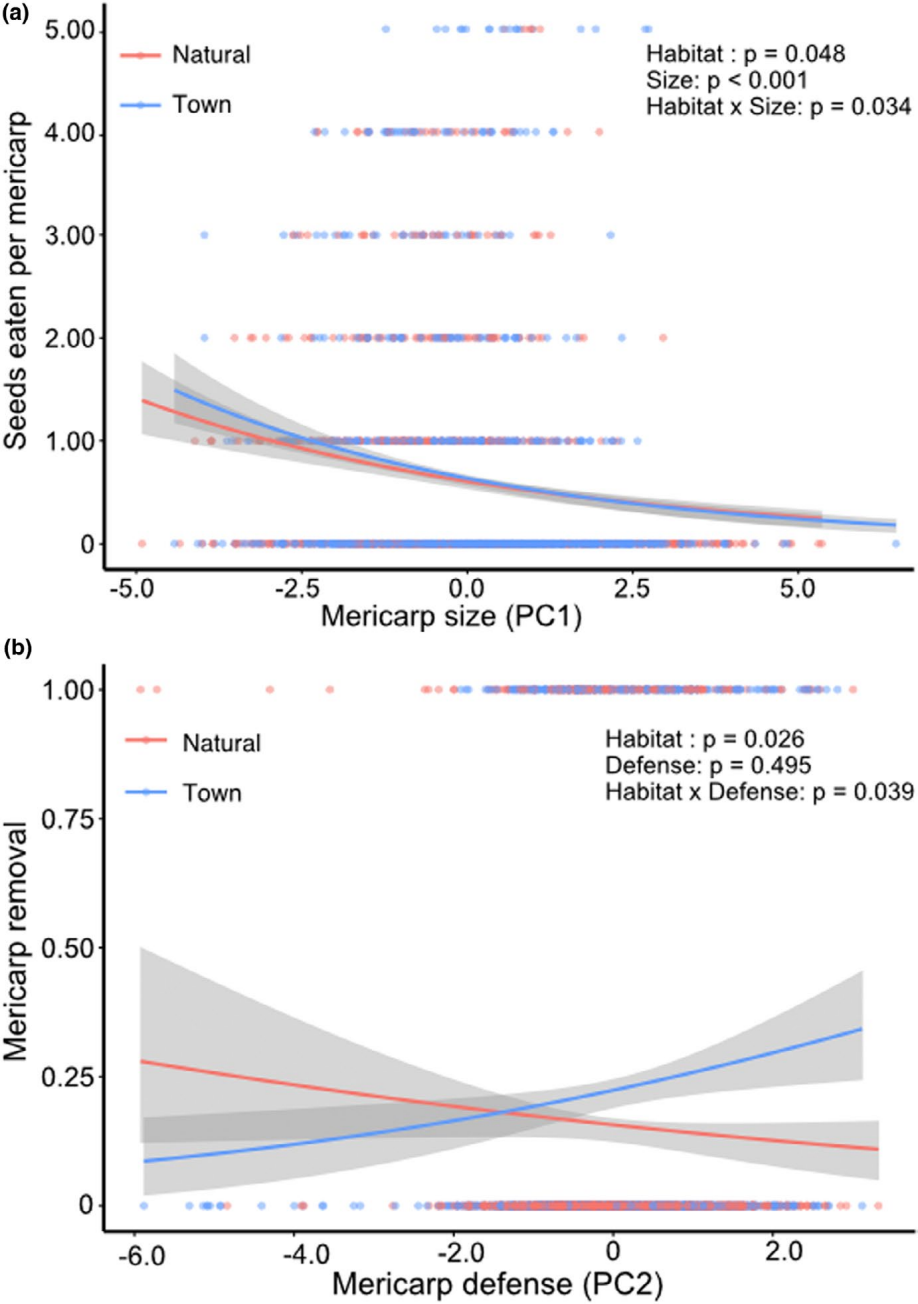
Seed and fruit removal by finches in natural and experimental populations of *Tribulus cistoides*. (a) The number of seeds eaten per mericarp declined with cumulative mericarp size (PC1) in natural habitats of *T*. *cistoides*, with small mericarps being eaten more in town habitats than in natural habitats. (b) Mericarp removal (1 = mericarp removed; 0 = mericarp remained) from experimental populations increased with mericarp defense (PC2) in town habitats but declined in natural habitats. Shading around the lines represents the 95% confidence errors of the model

There were effects of habitat, mericarp size, and defense on the seed removal rate in *T*. *cistoides* populations (Table 1). The number of seeds eaten per mericarp was 1.25% higher in towns than natural habitats (*Habitat*: $\chi_{1}^{2}$ = 3.91, *p* = .048). Overall, more seeds were eaten from small mericarps (*Size*: $\chi_{1}^{2}$ = 10.74, *p* < .001), and seed removal from small mericarps was greater in towns than in natural habitats (*Habitat* × *Size*: $\chi_{1}^{2}$ = 4.51, *p* = .034; Figure 2a), suggesting that seed removal imposed stronger phenotypic selection against small mericarps in towns than in natural habitats. There was no main effects of island or defense; however, the effect of both size and defense differed among islands *(Island* × *Size*: $\chi_{2}^{2}$ = 8.89, *p* = .012; *Island* × *Defense*: $\chi_{2}^{2}$ = 6.69, *p* = .035), consistent with phenotypic selection on these traits varying among islands. When we reran the model with individual size traits (length, width, and depth) as covariates, we found that the effect of mericarp width on predation differed between habitats (*Habitat* × *Width*: $\chi_{1}^{2}$ = 7.49, *p* = .017), but found no effect of mericarp length or depth. Lastly, we found that mericarp size and defense traits differed between habitats and among islands (Table S2). Mericarps were 7% longer (*Habitat*: $\chi_{1}^{2}$ = 11.48, *p* = .002), and spines were more likely to be present (*Habitat*: $\chi_{1}^{2}$ = 9.41, *p* = .006), in towns than natural habitats. We also found the spines were 3% longer in towns, although this effect was marginally significant following the *p*‐value correction for multiple tests (*Habitat*: $\chi_{1}^{2}$ = 4.93, *p* = .079), but we found no effect of habitat on any of the other morphological traits.

**TABLE 1:** Results from the population survey and mericarp removal experiment across three islands in the Galápagos

	df	(A) Seeds eaten per habitat		(B) Mericarp removal	
		*χ* ^2^	*p*‐value	*χ* ^2^	*p*
Habitat	1	3.91	.**048**	4.98	.**026**
Island	2	3.98	.137	2.91	.234
Size	1	10.74	**<.001**	2.40	.121
Defense	1	3.13	.077	0.46	.495
Clipped	1	NA	NA	8.44	.**004**
Habitat × Island	2	5.77	.056	3.28	.194
Habitat × Size	1	4.51	.**034**	2.25	.134
Habitat × Defense	1	2.51	.113	4.24	.**039**
Habitat × Clipped	1	NA	NA	0.05	.823
Island × Size	2	8.89	.**012**	5.74	.057
Island × Defense	2	6.69	.**035**	2.46	.292

Note

We examined two response variables: the number of seeds eaten per mericarp (population survey results; *N* = 1699 mericarps) and the proportion of mericarps which were removed from a habitat (experimental results; *N* = 2120 mericarps). Results presented in the table show the fixed effects of habitat, island, mericarp size and defense, spine clipping (experimental results only), and their interactions. The significance of fixed effects was estimated with Wald *χ* ^2^ test statistics using Type III sums‐of‐squares.

### 3.2 Fruit removal experiment

Mericarp removal during our experiment was influenced by habitat and defense traits (Table 1). The mericarp removal was 43% higher in town populations (*Habitat*: $\chi_{1}^{2}$ = 4.98, *p* = .026) and 39% higher on clipped (undefended) mericarps (*Clipped*: $\chi_{1}^{2}$ = 8.44, *p* = .004), although this effect did not vary with habitat (*Habitat* × *Clipped*: $\chi_{1}^{2}$ = 0.05, *p* = .823). However, habitat influenced the effect of natural variation in mericarp defenses on predation (*Habitat* × *Defense*: $\chi_{1}^{2}$ = 4.24, *p* = .039). Well‐defended mericarps were removed more often from towns than natural habitats (Figure 2b), suggesting that fruit removal by finches selects against well‐defended mericarps in towns. In contrast, mericarps that were poorly defended experienced higher removal rates in natural populations than in town populations, indicating that the defensive function of spines completely switched between town and natural habitats (Figure 2b). When we ran the models with individual defense traits as covariates instead of the composite defense trait, we found that mericarps with longer spines were more likely to be removed in town habitats than natural habitats (*Habitat* × *Spine length*: $\chi_{1}^{2}$ = 4.95, *p* = .027), but there was no effect of lower spine or spine position.

### 3.3 Finch community composition

We detected an effect of habitat on total finch number, diversity, and community composition. We observed five species of finches across habitats: two *G*. *magnirostris*, 171 *G*. *fortis*, 268 *G*. *fuliginosa*, 54 *G*. *scandens*, and 11 *Platyspiza crassirostris*. We found no change in the total or relative counts of *G*. *fortis* in the finch communities among town and natural habitats (*Habitat*: $\chi_{1}^{2}$ = 1.10, *p* = .294) or among islands (*Islands*: $\chi_{2}^{2}$ = 1.22, *p* = .544). We were unable to evaluate differences in the number of *G*. *magnirostris* among populations because only two individuals were observed, both in towns.

Finch counts were 17% lower in towns than natural habitats (*Habitat*: $\chi_{1}^{2}$ = 5.37, *p* = .017; Table 2), and these effects differed between islands (*Habitat* × *Island*: $\chi_{1}^{2}$ = 6.94, *p* = .031; Table 2). Finch counts were 55% and 18% higher in natural habitats relative to towns on Isabela and Santa Cruz, respectively, whereas there was no difference in counts between habitats on Floreana. The effects of habitat on diversity also differed similarly among islands (*Habitat* × *Island*: *F*_1_ = 8.591, *p* = .001; Table 2).

**TABLE 2:** Results from finch surveys across islands (*N* = 60 observations) for counts, Shannon Diversity Index, and community composition

Response	Predictor	df	*χ* ^2^/*F*‐value	*p*
Count	Habitat	1	5.67	.**017**
	Island	2	3.86	.145
	Population	5	15.84	.**007**
	Habitat × Island	2	6.94	.**031**
Diversity	Habitat	1	1.49	.229
	Island	2	3.30	.**045**
	Population	5	1.47	.218
	Habitat × Island	2	8.59	.**001**
Community	Habitat	1	8.49	**<.001**
Composition	Island	2	4.18	.**002**
(RDA)	Population	5	0.41	.971
	Habitat × Island	2	2.71	.**023**

Note

Results presented in the table show the fixed effects of habitat, island, population, and the interaction between habitat and island. The significance of fixed effects for abundance were estimated using Wald *χ* ^2^ test statistics with Type III sums‐of‐squares. The significance of fixed effects for diversity and community composition were estimates with pseudo‐*F* test statistics.

The results from the RDA were consistent with the results for abundance and diversity (Figure S2; Table 2). The composition of the finch community differed between towns and natural habitats (Habitat: *F*_1,51_ = 8.49, *p* < .001) and among islands (*Island*: *F*_2,51_ = 4.18, *p* < .001), as did the effect of habitat among islands (*Habitat* × *Island*: *F*_2,51_ = 2.71, *p* = .023). Although communities somewhat overlapped across populations, small ground finches (*G*. *fuliginosa*) were more frequently observed in towns than in natural habitats.

## 4 DISCUSSION

We found that towns modified interactions between Darwin's finches and *T*. *cistoides*. Seed and fruit removal rates were higher in towns in both surveyed and experimental populations (Q1), and towns exhibited modified phenotypic selection on mericarp morphology (Q2). In the surveyed populations, seed removal imposed stronger phenotypic selection against small mericarps in towns than in natural habitats, while in experimental populations fruit removal selected against well‐defended mericarps in towns and poorly defended mericarps in natural habitats. Lastly, while we found no difference in the number of *G*. *fortis* and *G*. *magnirostris*, towns supported smaller and less diverse ground finch communities (Q3). Together, our results suggest even the earliest stages of urbanization can alter ecological interactions between finches and *T*. *cistoides*, leading to modified phenotypic selection and phenotypic trait differences in *T*. *cistoides* populations.

### 4.1 Mericarp predation in towns

We observed direct effects of habitat on seed and mericarp removal. We found that predation was higher in towns than natural habitats in both the population surveys and our experiment. Increased predation in towns is consistent with the hypothesis that urbanization intensifies interactions between finches and *T*. *cistoides*. Interaction strength may have increased because *T*. *cistoides* is more abundant in towns on the Galápagos islands (M.T.J.J. and R.A.J., personal observation). Humans have been shown to be unintentional dispersers of *T*. *cistoides* (Johnson et al., 2020), making *T*. *cistoides* populations more likely to be established in and around towns. If finches exhibit a functional response to *T*. *cistoides*, then their consumption of *T*. *cistoides* seeds may be correlated with the plant's abundance in a habitat (Abrams, 1982). This functional response would explain why *T*. *cistoides* populations in towns experienced greater predation from foraging finches, despite fewer finches being observed in these environments.

### 4.2 Phenotypic selection on mericarp morphology

We found that towns altered phenotypic selection on mericarp morphology, where finch predation in towns selected against small and well‐defended mericarps. In the population survey, we observed greater seed removal from small mericarps in all environments, but this effect was strongest in towns. Small mericarps may be more energetically efficient to open (Grant & Grant, 2006; Price, 1987), leading finches to choose small mericarps over large ones. Combined with greater abundances of *T*. *cistoides* in towns, preferential consumption of small mericarps may intensify phenotypic selection in favour of large mericarps in towns. Such selection may be absent in natural populations if finches have access to other food resources aside from *T*. *cistoides*. Our finding that mericarps are longer in towns is consistent with the hypothesis that towns impose phenotypic selection against small mericarps, potentially leading to the evolution of larger fruits, although our current design does not allow us to distinguish between evolved versus plastic responses. Our results contrast with a previous study that found that finches select for smaller mericarps in natural populations of *T*. *cistoides* (Carvajal‐Endara et al., 2020). The differences in findings may be the result of yearly variation in climate that contributes to differences in resource availability (Siepielski et al., 2017) and thus differences in the intensity of mericarp consumption by finches.

In the experimental populations, we observed increased removal of well‐defended mericarps from town habitats, whereas we observed the opposite trend in natural habitats. This result suggests that urbanization may be associated with finches preferring better defended mericarps. It is presently unclear why urbanization modifies the direction of phenotypic selection on mericarp defense. Mericarp spines are expected to deter predators from accessing the seeds, an expectation that is consistent with our findings from the natural habitats and those from Carvajal‐Endara et al. (2020). However, we found that in towns, finches preferred mericarps with longer spines, perhaps because they were easier to pick up and manipulate. Additionally, the population surveys found that mericarps had longer spines and lower spines more likely to be present in towns, which is not consistent with phenotypic against well‐defended fruits in towns. Further experiments that include finch observations at each experimental site and concrete measures of evolved versus plastic responses (e.g., reciprocal transplants between habitats) are needed to untangle these findings.

Our survey and experiment were independent studies that capture variation in finch behaviour. While our experiment was complementary to our population survey, these two components of our study differed in several important ways. First, we surveyed established populations that were available to finches for longer than the experimental populations, potentially leading to variation in seed removal unrelated to mericarp morphology in the surveyed populations. In addition, surveyed populations may have been more likely to experience seed removal because finches would already have known where to find them, whereas finches had to first locate the novel experimental populations before removing the mericarps. Surveyed populations also experienced a greater range of climatic variation, potentially affecting the strength of phenotypic selection they experienced (Siepielski et al., 2017). The shorter‐term nature of the experiment also meant that the experimental populations experienced a shorter window of predation pressure than the surveyed populations. Lastly, we were unable to track how many seeds were removed from each mericarp in the experimental populations. Seed removal gives a more precise estimate of the fitness effects experienced by the plants and could explain the different patterns of phenotypic selection between the surveyed and experimental populations.

### 4.3 Urban finch communities

Differences in the finch communities between the towns and natural habitats may have contributed to patterns of predation and phenotypic selection i/n *T*. *cistoides*. Finch counts were lower in towns relative to natural habitats, although this effect varied among islands. We observed smaller and less diverse finch communities in the largest two towns in our study. These towns are also undergoing rapid human population expansion (Figure 1B), and the patterns of finch abundance on these islands are consistent with other studies that find that bird communities are often negatively affected by urbanization (reviewed in Aronson et al., 2016). Our findings support the hypothesis that urbanization acts as a selective filter on species communities, likely contributing to altered interactions between finches and *T*. *cistoides*. In contrast, there were no significant differences in number or diversity of finches between habitats on Floreana, the smallest and least developed island, suggesting that town size and stage of development play a role in shaping finch communities. Our study is one of several to identify an impact of human disturbance on the ecology of Darwin's finches in the Galápagos. Urbanization reduces antipredator behaviour in finches, despite elevated predation risk from human pets on certain islands (Gotanda, 2020). Urbanization also decreases resource partitioning through relaxed selection on beak morphology in *G*. *fortis*, a trend that is most likely explained by increased food availability from anthropogenic food subsidies (De León et al., 2019; Hendry et al., 2006). Human food subsidies have been shown to alter the gut microbiota in urban finch communities (Knutie et al., 2019) and may also be linked to the epigenetic changes associated with urbanization (McNew et al., 2017). While our study tracked how consumption by finches affects ecological and evolutionary processes on *T*. *cistoides*, it would be interesting to determine if an evolutionary response of *T*. *cistoides* populations feedbacks to affect the ecology and evolution of finches.

## 5 CONCLUSIONS

Together, our results suggest that moderate urban development can modify interactions between Darwin's finches and *T*. *cistoides* on the Galápagos Islands. Islands are predicted to be particularly sensitive to anthropogenic disturbance, and perturbations to the landscape through urban development, increased human activity, and the introduction of invasive species may have large‐scale effects on the ecology and evolution of native island species (Helmus et al., 2014). Our study is one of the first to identify urbanization as a key mediator of the evolutionary ecology of species interactions on tropical islands.

## CONFLICT OF INTEREST

None declared.

## AUTHOR CONTRIBUTIONS

**L. Ruth Rivkin:** Data curation (lead); formal analysis (lead); validation (lead); writing–original draft (lead); writing–review and editing (lead). **Reagan A. Johnson:** Investigation (equal); methodology (equal); project administration (equal); writing–original draft (supporting). **Jaime A. Chaves:** Funding acquisition (equal); investigation (supporting); resources (equal); writing–original draft (supporting). **Marc Johnson:** Conceptualization (lead); data curation (equal); funding acquisition (equal); investigation (equal); methodology (equal); project administration (lead); supervision (lead); writing–original draft (supporting); writing–review and editing (supporting).

## Supporting information

Appendix S1

## Data Availability

Data and code are archived on Dryad: https://doi.org/10.5061/dryad.j9kd51cdd.
