# Supplementary material for: Urbanization alters interactions between Darwin's finches and Tribulus cistoides on the Galápagos Islands
Source: Ecol Evol. 2021 Oct 26;11(22):15754–65. doi: 10.1002/ece3.8236 (PMC8601916; doi:10.1002/ece3.8236)
Supplement: Supplementary file 1 — Appendix S1 [file ECE3-11-15754-s001.docx]

**Urbanization alters interactions between Darwin’s finches and *Tribulus cistoides* on the Galápagos Islands**

L. Ruth Rivkin, Reagan A. Johnson, Jaime A. Chaves, Marc T.J. Johnson

**Supplemental Tables**

Table S1: Proportion of variance explained by each PC axis for size and defense traits of mericarps from the surveyed populations and experimental populations.

|  | Proportion of Variance | |
| --- | --- | --- |
|  | Surveyed populations | Experimental populations |
| PC1 | 0.4472 | 0.4558 |
| PC2 | 0.2005 | 0.1700 |
| PC3 | 0.1660 | 0.1416 |
| PC4 | 0.0899 | 0.1213 |
| PC5 | 0.0647 | 0.0668 |
| PC6 | 0.0317 | 0.0297 |

Table S2: Results from mericarp trait differences among habitats and islands, estimated from the population surveys conducted three islands in the Galápagos. Results presented in the table show the fixed effects of habitat and island. The significance of fixed effects were estimated with Wald χ^2^ test statistics using Type III sums-of-squares, and p-values have been corrected for multiple tests. Significant p-values have been bolded

| Response | Predictor | df | χ^2^/F-value | *P* |
| --- | --- | --- | --- | --- |
| Length | Habitat | 1 | 11.45 | **0.002** |
|  | Island | 2 | 75.55 | **< 0.001** |
| Width | Habitat | 1 | 1.18 | 0.831 |
|  | Island | 2 | 23.48 | **< 0.001** |
| Depth | Habitat | 1 | 0.02 | 1.00 |
|  | Island | 2 | 62.64 | **< 0.001** |
| Spine length | Habitat | 1 | 4.93 | 0.079 |
|  | Island | 2 | 61.51 | **< 0.001** |
| Lower spine | Habitat | 1 | 9.41 | **0.006** |
|  | Island | 2 | 66.34 | **< 0.001** |
| Spine position | Habitat | 1 | 0.44 | 0.775 |
|  | Island | 2 | 0.01 | 0.996 |
|  |  |  |  |  |
|  |  |  |  |  |

**Supplemental Figures**


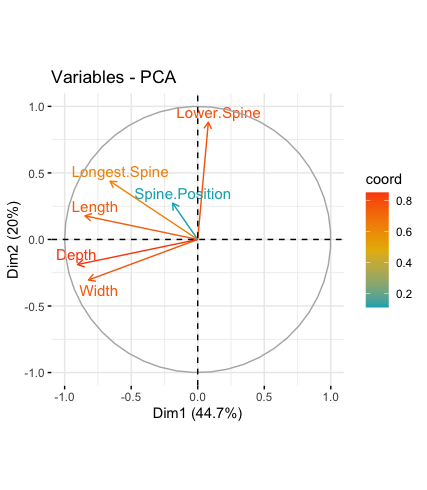

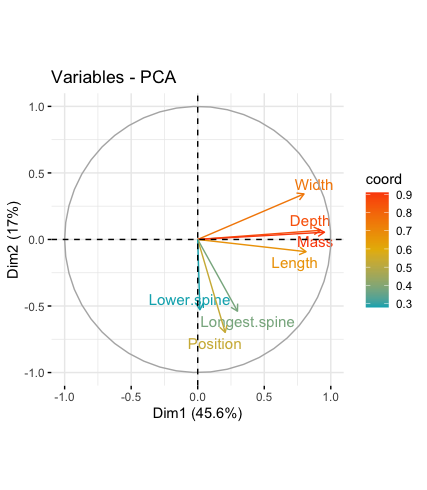


a

b

Figure S1: Loadings of size and defense traits of mericarps from (a) surveyed and (b) experimental populations. Each variable is color-coded by their coordinates of the individuals in the PCA.


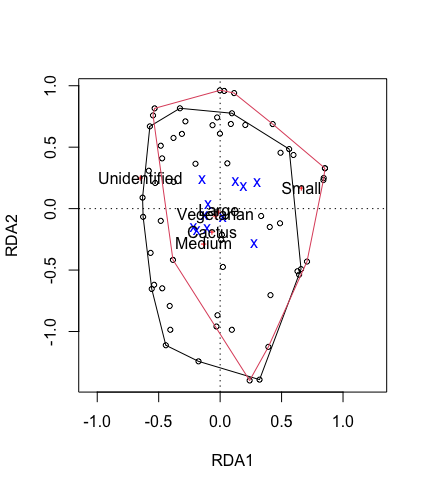


Figure S2: Community composition plot of town (red) and natural (black) finches across the three islands in our study.
